# Recent status and trends of nanotechnology in cervical cancer: a systematic review and bibliometric analysis

**DOI:** 10.3389/fonc.2024.1327851

**Published:** 2024-02-20

**Authors:** Xiangzhi Song, Xun Li, Zhiwei Tan, Lushun Zhang

**Affiliations:** ^1^ School of Clinical Medicine, Chengdu Medical College, Chengdu, China; ^2^ Department of Pathology, The Second Affiliated Hospital of Chengdu Medical College, China National Nuclear Corporation 416 Hospital, Chengdu, Sichuan, China; ^3^ Development and Regeneration Key Laboratory of Sichuan Province, Department of Neurobiology, Chengdu Medical College, Chengdu, China; ^4^ Department of Pathology and Pathophysiology, Chengdu Medical College, Chengdu, China

**Keywords:** nanotechnology, cervical cancer, bibliometrics, nanoparticles, drug deliver

## Abstract

**Background:**

Cervical cancer is currently the second leading cause of cancer death among women from developing countries (1). However, there is a lack of effective treatment methods, and the existing treatments often result in significant adverse reactions and high chances of recurrence, which ultimately impact the prognosis of patients. As a result, the application of nanotechnology, specifically nanoparticle-based approaches, in the diagnosis and treatment of cervical cancer has gained significant attention. This study aims to examine the current research status and future development trends of nanotechnology in relation to cervical cancer using a bibliometric perspective.

**Methods:**

A bibliometric analysis was performed to gather relevant research papers from the Web of Science database. VOSviewer and CiteSpace were utilized to conduct quantitative analysis and identify hot topics in the field, focusing on countries, institutions, journals, authors, and keywords.

**Result:**

A total of 997 eligible literature were retrieved. From January 1, 2014 to September 20, 2023, the overall number of publications showed an upward trend. The paper mainly comes from China (n=414). The main institution is the Chinese Academy of Sciences (n=62), and 60% of the top 10 institutions in the number of documents issued are from China. First authors Ma, Rong (n=12) and Alifu, Nuernisha (n=12). The journal with the highest publication volume is ACS Applied Materials&INTERFACES (n=35), and the journal with the highest citation frequency is BIOMATERIALS (n=508). “Nanoparticles (n=295)”, “cervical cancer (n=248)”, and “drug delivery (n=218)” are the top three most frequently occurring keywords. In recent years, photothermal therapy and indocyanine green have become research hotspots.

**Conclusion:**

The application of nanotechnology in the field of cervical cancer has garnered considerable attention. Nanoparticles-based methods for diagnosis, administration, and treatment have proven to be instrumental in enhancing the sensitivity of cervical cancer detection, improving the accuracy and efficiency of administration, and reducing drug toxicity. Enhancing treatment efficacy and improving patient prognosis have emerged as current research priorities and future directions.

## 1 Introduction

Globally, cervical cancer is the fourth most common type of cancer among women and the second leading cause of cancer-related deaths among women from developing countries (1, 2). The majority of cervical cancers are caused by human papillomavirus (HPV), a virus similar to the human immunodeficiency virus. HPV poses the highest risk for cervical cancer and can only be managed through a limited number of highly invasive methods (2, 3). While chemotherapy and surgery have been proven highly effective in treating early-stage cervical cancer, surgery is still the preferred option due to potential damage to the female reproductive system caused by chemotherapy. Chemotherapy remains effective for those with more advanced conditions (4, 5). However, incomplete surgical resection, adverse reactions to chemotherapeutic agents, and cellular resistance to multiple drugs can lead to unsatisfactory long-term results. Additionally, the complex nature of tumor cells and the harsh tumor microenvironment make them resistant to treatment and metastasis, posing a significant obstacle to the possibility of recurrent cervical cancer. This could be due to remaining tumor cells or metastatic tumor cells (6, 7). Therefore, there is an urgent need for a more effective method to diagnose early-stage cervical carcinogenesis, improve the efficacy of drug action, reduce adverse effects, and enhance the quality of prognosis.

In comparison to other diagnostic modalities, various nanoparticle-based measurements in nanotechnology have demonstrated improved selectivity and sensitivity, as well as provided support for obtaining information that traditional measurements cannot. By leveraging the exceptional optical and electrical properties of nanomaterials, such as gold nanoparticles, along with their significantly larger surface area, they can be integrated with various types of sensors, including electrochemical sensors and DNA biosensors, to effectively detect biomarkers. This approach successfully addresses the limitations of traditional sensors, such as low conductivity, low sensitivity, and high cost (8–11). This enables early detection of tumor onset and timely intervention, ultimately aiming to enhance patient survival. Nanoparticles can also be utilized to monitor disease progression in patients, facilitating the formulation of more precise treatment strategies (12). Depending on the characteristics of the tumor microenvironment, such as hypoxia and acidity, nanoparticles can be designed to respond effectively in various environments, thereby enhancing treatment outcomes. By modifying the properties of nanoparticles, they can facilitate the uptake of drugs into the tumor tissue of the uterine cervix, increase their concentration in tumor tissue, and improve treatment efficacy. In addition, nanoparticles can be used as efficient carriers for drug delivery due to their good biocompatibility, permeability, and retention. Utilizing nanoparticles as delivery systems for anticancer agents can enhance drug efficacy, reduce dosing and treatment frequency, and potentially minimize the toxicity of antitumor drugs to the human body (13). With the deepening of research on nanotechnology, researchers have also designed some progressively stimuli-responsive drug delivery systems based on this foundation, which can enhance drug delivery more effectively (14). Furthermore, certain nanoparticles can be employed as specific markers targeting cancer cells (15). For example, using exosomes isolated from the tumors of cervical cancer patients as nanocarriers to transport drugs can robustly target the target cells (16). The development of nanotechnology has significantly enhanced various aspects of medicine, particularly in the field of cancer diagnosis and treatment, where improved diagnostic methods and novel drug delivery systems hold great promise (17). The combination of nanotechnology and cervical cancer research may yield unexpected advancements in the future.

Currently, there is no research analyzing the application of nanotechnology in cervical cancer using the bibliometrics approach. Bibliometrics involves analyzing published information, such as books, journal articles, and data sets, along with their associated metadata like abstracts, keywords, and citations. It also includes using statistical information to describe the relationship between published works. Tools like VOSviewer and CiteSpace can be used to visualize and analyze academic literature in this research field (18). By employing bibliometrics, we can gain a better understanding of existing research, identify current areas of interest, and predict future trends in nanotechnology for cervical cancer. The objective of this study is to conduct a comprehensive analysis of the trends of nanotechnology in cervical cancer from various angles using bibliometric tools. This analysis will identify the prominent countries, journals, institutions, and authors that have contributed to this field. Additionally, the study will focus on analyzing and discussing the current research areas of interest and potential future research directions. The findings will be beneficial for researchers seeking to gain a deeper understanding of this field or for those who are new to it.

## 2 Materials and methods

### 2.1 Data source and collection

The Web Science Core Collection (WoSCC) is a widely used bibliometric analysis database that contains over 10,000 high-quality journals and comprehensive citation records (19). Additionally, WoSCC has been found to have more accurate document type labeling compared to other databases (20, 21).

**Figure 1:**
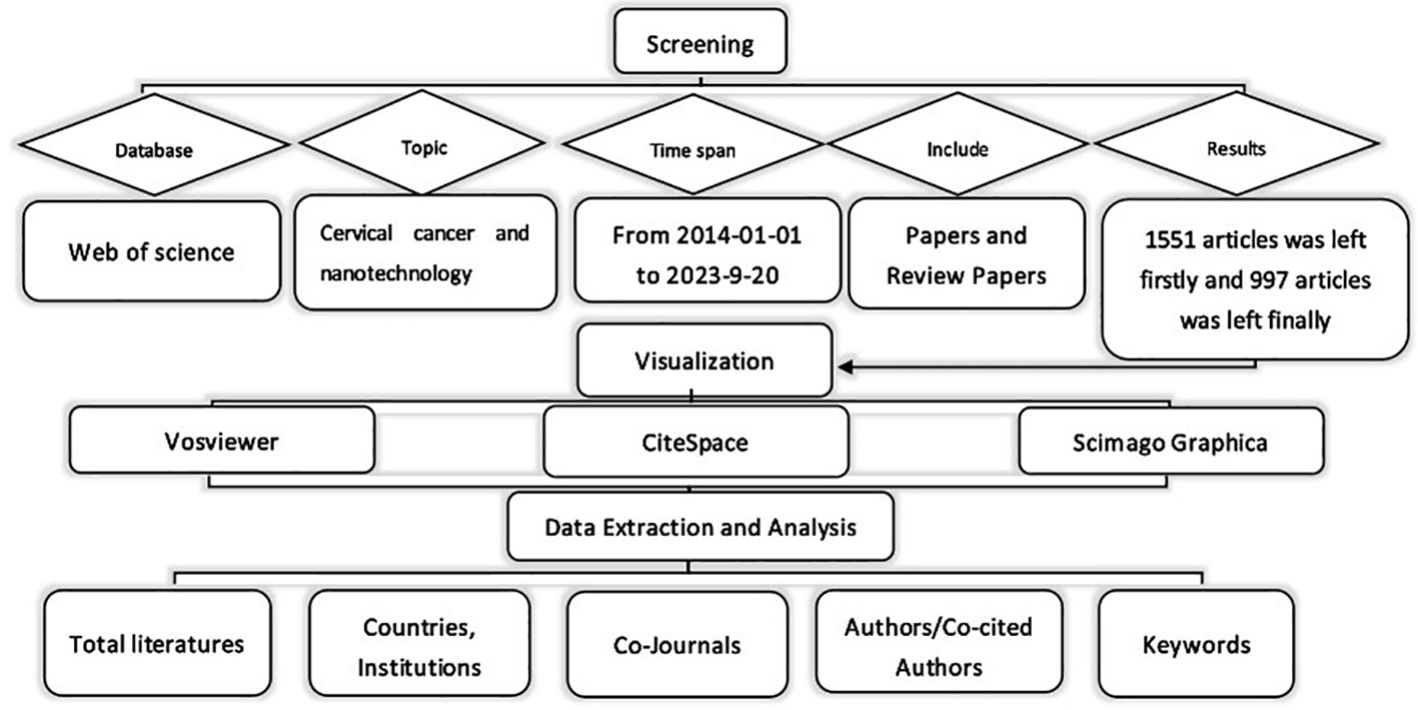
Flow chart of literature selection.

**Figure 1** illustrates the workflow for this study. In September 2023, we conducted a search for original research articles related to nanotechnology for cervical cancer that were published between January 1, 2014, and September 20, 2023. To ensure inclusivity and eliminate irrelevant articles, we designed a search strategy that involved the following steps: 1) Using keywords such as ‘cervical cancer’ and ‘nanotechnology’; 2) Including various synonyms for these keywords, including specific names of nanomaterials and nanoparticles; 3) Excluding papers that were not original research; 4) Limiting the search to English publications only; 5) Focusing on the data categories of ‘articles’ and ‘reviews’. After applying these criteria, we evaluated the title and abstract of each article to determine its relevance to the topic. In cases where the literature was inconclusive, two researchers read the full text and conducted a more detailed evaluation to ensure accurate results. Subsequently, we identified pertinent articles and extracted primary data on nations/regions, institutions, authors, journals, and key terms. For the specific search formula used in this article, please refer to **Supplementary Materials 1**.

### 2.2 Data analysis and visualization

The collected literature was analyzed using CiteSpace, which provided insights into authors, countries, institutions, journals, and keywords. To visualize keyword co-occurrence, VOSviewer was employed, representing authors or keywords as nodes with varying sizes based on their frequency. The strength of connections was depicted by line thickness, while node colors indicated different clusters or periods. The Scimago Graphica tool was utilized to visualize the overall distribution of publications.

## 3 Results

### 3.1 Annual publications and trends

**Figure 2:**
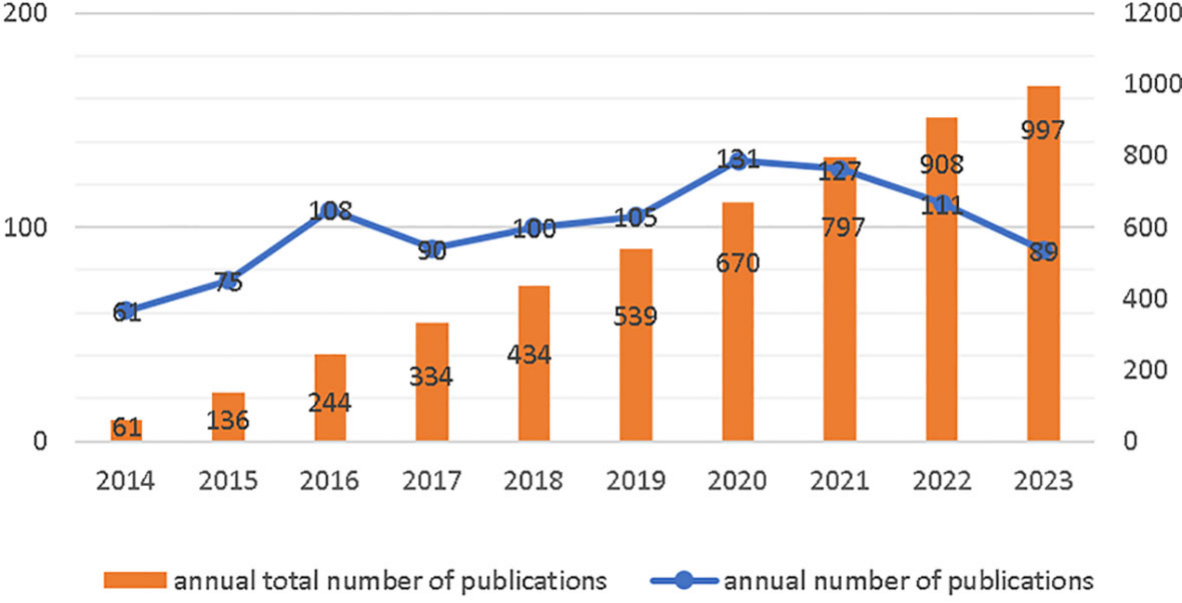
Annual Publications and Trends.

To some extent, the number of publications can represent the heat and development of the field in a given time. From January 1, 2014 to September 20, 2023, WOS received a total of 997 publications on nanotechnology in cervical cancer research, including 941 articles and 56 reviews. **Figure 2** shows the worldwide trend in number of publications per year and total number of publications for research in cervical cancer for the period January 2014 to September 2023. Each year of the decade, the number of articles published was close to 100, with occasional declines in the intervening years, with an average annual growth rate of 2.39%.

### 3.2 Contributions of authors

There were 5566 authors included in the study, with the **Table 1** showing the top ten authors and co-citations. Ma, Rong (n=12) and Alifu, Nuernisha (n=12) had the highest number of articles published, followed by Yan, Ting (n=11), Alimu, Gulinigaer (n=11). The CiteSpace was used to show the referencing relationships between different authors (**Supplementary Material Figure 1A**). Nodal sizes represent the number of papers, and rows represent connections between authors. Co-citation means that two or more authors are cited simultaneously in one or more articles, and that these two or more authors form a co-citation relationship. Overall, 27787 authors were cited at least once (data obtained from VOSviewer). WANG Y (n=73) is the most cited author of the network built by CiteSpace, LIU Y is the author with the highest centrality (0.18), ranking fifth in terms of publication volume and considerable output (The network of co-cited author relationships can be found in the **Supplementary Material Figure 1B**).

**Table 1:** Contributions of authors.

Rank	Author	Documents	Burst	Co-cited Author	Documents	Centrality
1	Ma, Rong	12	3.59	WANG Y	73	0.13
2	Alifu, Nuernisha	12	3.59	ZHANG Y	71	0.14
3	Yan, Ting	11	3.26	ZHANG L	54	0.11
4	Alimu, Gulinigaer	11	3.26	LI L	52	0.14
5	Zhu, Lijun	10	4.16	LIU Y	50	0.18
6	Du, Zhong	10	2.96	WANG L	47	0.09
7	Zhang, Xueliang	10	4.16	WANG J	45	0.15
8	Xie, Zhigang	9	0	MAEDA H	44	0.05
9	Liu, Shi	9	0	CHEN Y	41	0.14
10	Chen, Shuang	9	3.74	FERLAY J	41	0.09

Top ten authors and co-citing authors of studies on nanotechnology and cervical cancer.

### 3.3 Contributions of countries or regions

**Figure 3:**
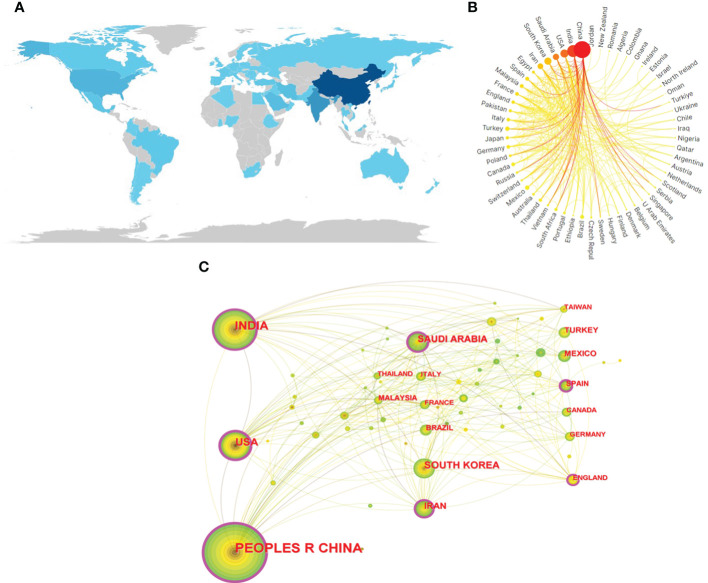
**(A)** Geographical distribution map based on the total publications of different countries/regions. **(B)** The international collaborations’ visualization map of countries/regions. **(C)** The countries/regions’ citation network visualization map was generated by using Citespace. The size of the circle in the visualization diagram built by citespace represents the number of papers published,and the thickness of the purple ring of the node represents the size of the center value.

Research on nanotechnology/nanomaterials in cervical cancer has been conducted in 164 countries/regions over the last decade. On the country distribution map (**Figure 3A**, the darker the color of the country, the more publications there are.), the top three countries/regions are China (n = 414,41.52%), India (n = 208, 20.86%) and the United States (n = 93, 9.32%) on the basis of the number of publications. The cooperation between many countries can be seen in **Figure 3B**, with the cooperation between China and India being the most evident. We then conducted a more in-depth analysis of high productivity countries in the field, and publications from the top 10 countries are shown in **Table 2** and **Figure 3C**. China was the country with the most publications, the United States had the highest average number of citations with a total of 33.13 citations. The degree of centrality is an assessment of the importance of the nodes in the network, primarily used to measure the value of nodes’ bridge functions across the network structure. The purple circles outside each node in **Figure 3C** represent the centrality of each country. Among the top 10 countries in the world, the United States has the highest betweenness centrality of 0.34, followed by China (0.25). Although Spain (0.23) did not have a large number of publications, their central values are high. Which shows that they play an essential role in international co-operation. The size of the circle in the visualization diagram built by CiteSpace represents the number of papers published, the thick line indicates cooperation between countries/regions and the width of the purple node ring represents the size of the central value.

**Table 2:** Contributions of countries/regions.

Rank	Countries/regions	Count	Centrality	Citations	Average Citations/Publications
1	CHINA	414	0.25	10723	25.90
2	INDIA	208	0.13	4818	23.16
3	USA	93	0.34	3081	33.13
4	SOUTH KOREA	73	0.01	2249	30.81
5	SAUDI ARABIA	56	0.13	1247	22.27
6	IRAN	47	0.16	1131	24.06
7	TURKEY	27	0.06	477	17.67
8	MEXICO	27	0.05	364	13.48
9	SPAIN	23	0.23	514	22.35
10	BRAZIL	22	0.02	426	19.36

Top ten countries/regions for related publications.

### 3.4 Contributions of institutions

Articles/comments received from the WOS are from 3229 different institutions. On the basis of the number of publications, **Table 3** comprises the top ten production agencies. It is not hard to see from table that six institutions are from China, with two institutions from India, one from Saudi Arabia and one from France. The United States has the highest number of citations and the third-highest number of publications, but none of the top ten publications are from it. The Chinese Academy of Sciences(n=62) has the largest number of publications and Indian Institute of Technology System (IIT System) has the highest centrality (0.2). The size of the circle in the visual map of CiteSpace (**Supplementary Material Figure 2**) represents the number of articles published by each institution, each row represents the collaboration between the two institutes, and the purple circle represents the node with a higher centrality, the higher the number, the greater the centrality. The top 10 universities published 239 articles, or 23.97% of the total, greatly promoted the development of fields related to the subject.

**Table 3:** Contributions of institutions.

Rank	Institutions	Count	Centrality	Country
1	Chinese Academy of Sciences	62	0.12	China
2	Indian Institute of Technology System (IIT System)	47	0.2	India
3	Changchun Institute of Applied Chemistry	24	0.03	China
4	King Saud University	19	0.14	Saudi Arabia
5	University of Chinese Academy of Sciences	17	0.1	China
6	Indian Institute of Science Education & Research (IISER) Pune	16	0.01	India
7	Xinjiang Medical University	15	0.04	China
8	Jilin University	14	0	China
9	Centre National de la Recherche Scientifique (CNRS)	13	0.09	France
10	Zhejiang University	12	0.01	China

Top ten institutions for related publications.

### 3.5 Analysis of journals/co-cited journals

332 journals have published papers on nanotechnology and hepatic cancer, of which ACS APPLIED MATERIALS & INTERFACES (n = 35, IF 2022 = 9.5) ranks first and JOURNAL OF MATERIALS CHEMISTRY B (n =34, IF 2022 = 7) ranked second. RSC ADVANCES (n = 32, IF 2022 = 3.9) ranked third. Of the top ten journals, 30% (3/10) are from Netherlands and 30% (3/10) are from the England. JOURNAL OF CONTROLLED RELEASE (n = 18, IF 2022 = 10.8) was the journal with the largest impact factor. The details are shown in **Table 4A**.

**Table 4A:** Journal analysis.

Rank	Journals	Documents	IF2022	JCR	Country
1	ACS APPLIED MATERIALS & INTERFACES	35	9.5	Q1	UNITED STATES
2	JOURNAL OF MATERIALS CHEMISTRY B	34	7	Q1	ENGLAND
3	RSC ADVANCES	32	3.9	Q2	ENGLAND
4	COLLOIDS AND SURFACES B-BIOINTERFACES	26	5.8	Q1	NETHERLANDS
5	INTERNATIONAL JOURNAL OF NANOMEDICINE	19	8	Q1	NEW ZEALAND
6	NANOSCALE	19	6.7	Q1	ENGLAND
7	JOURNAL OF CONTROLLED RELEASE	18	10.8	Q1	NETHERLANDS
8	INTERNATIONAL JOURNAL OF BIOLOGICAL MACROMOLECULES	17	8.2	Q1	NETHERLANDS
9	INTERNATIONAL JOURNAL OF MOLECULAR SCIENCES	13	5.6	Q1	UNITED STATES
10	SCIENTIFIC REPORTS	13	4.6	Q2	ENGLAND

Top ten journals for related publications.

Among the co-cited journals, BIOMATERIALS (n = 508, IF 2022 = 14) ranked first, ACS NANO (n = 440, IF 2022 = 17.1) ranked second. J CONTROL RELEASE (n = 419, IF 2022 = 3.9) was ranked third but had the largest centrality. The details are shown in **Table 4B**.

**Table 4B:** Journal analysis.

Rank	Co-cited journals	Count	Centrality	IF2022	JCR
1	BIOMATERIALS	508	0.03	14	Q1
2	ACS NANO	440	0.02	17.1	Q1
3	J CONTROL RELEASE	419	0.03	3.9	Q2
4	INT J NANOMED	393	0.01	8	Q2
5	ADV DRUG DELIVER REV	357	0.02	16.1	Q1
6	ACS APPL MATER INTER	352	0.01	9.5	Q2
7	J AM CHEM SOC	344	0.01	15	Q1
8	NANOSCALE	343	0.01	6.7	Q2
9	COLLOID SURFACE B	332	0.01	5.8	Q1
10	ADV MATER	293	0.01	29.4	Q1

Top ten co-cited journals for related publications.

### 3.6 Analysis of keywords

**Figure 4:**
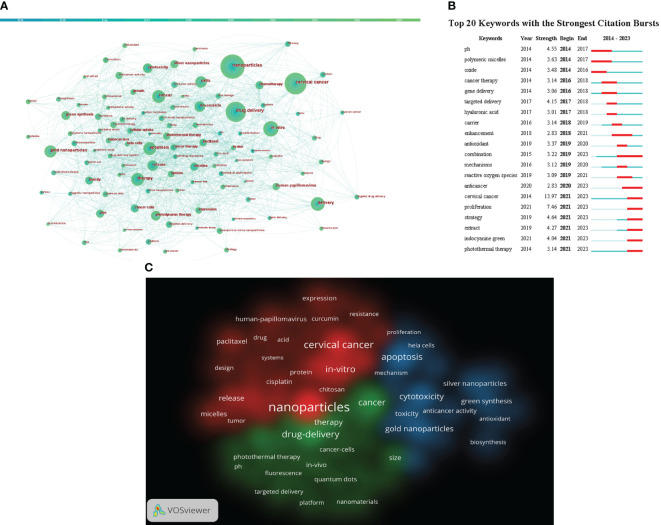
**(A)** Citespace visualization map of keywords. **(B)** CiteSpace visualization map of the top 20 keywords for the strongest citation bursts from January 2014 to September 2023 **(C)** VOSviewer visualization map of Cluster view of keywords. Different colors indicate different clusters. (Source: https://www.vosviewer.com/).

Keywords condense the heart and essence of the paper, and through analysis of the co-occurrence of keywords can reveal the research hotspot of a certain scientific field. For example, in a visual network constructed using CiteSpace (**Figure 4A**), the larger the circular node, the greater the number of keywords, and the more representative the hotspot. The nodal line indicates the strength of the association, and the thicker the nodal line, the stronger the connection between them. The highest frequency was observed in nanoparticles (n=295), followed by cervical cancer (n=248) and drug-delivery (n=218) as detailed in **Supplementary Material Table 1**.

The shortest outbreak duration based on the keyword outbreak map is 3 years, citing the top 25 keywords with the highest outbreak intensity (Details can be found in **Figure 4B**). From **Figure 4B**, keywords such as “oxide (2014–2016)”, “polymeric micelles (2014-1017)” and “gene delivery (2016-2018)” have been given more attention in the past. “extract (2021-2023)”, “indocyanine green (2021-2023)” and “photothermal therapy (2021-2023)” have received widespread attention recently. It may be a new direction for future research.

We used VOSviewer to create a view of the keyword co-occurrence network for 4250 documents and select 82 keywords that have a frequency greater than or equal to 20 for visualization (Details can be found in **Figure 4C**). Each of the three colors in the picture represents a different cluster, referred to as a research topic. In addition, in the figure, we find that the identified keywords are divided into three clusters: red, green and blue. Red indicates clustering keywords related to drug, including nanoparticles, in-vitro, cisplatin, paclitaxel, etc. The keywords of the green group are primarily bound up with the drug-delivery, including photothermal therapy, fluorescence, targeted delivery, etc. The blue areas are mainly related to cytotoxicity, including gold nanoparticles,silver nanoparticles, green synthesis and so on.

## 4 Discussion

### 4.1 General information

The number of annual publications and the total number of publications are both increasing, indicating a growing interest in the application of nanotechnology in cervical cancer research. The involvement of researchers in this field is also on the rise.

In terms of the number of articles published, China, India, and the United States ranked in the top three, suggesting their overall strength in this area. Among the major publishers, six out of the top ten are from China. The institutions with the highest number of published articles are CAS, Indian Institute of Technology System, and Changchun Institute of Applied Chemistry. However, global cooperation among institutions in this field remains uncommon. Future studies will explore the benefits of strengthening international cooperation between countries and institutions for the continued development of the field.

The majority of nanotechnology and cervical cancer-related journals are published in ACS APPLIED MATERIALS & INTERFACES, JOURNAL OF MATERIALS CHEMISTRY B, and RSC ADVANCES, with impact factors of 9.5, 7, and 3.9, respectively. BIOMATERIALS, ACS NANO, and J CONTROL RELEASE are the three most cited journals. These high-quality journals provide significant support for research in the field and indicate that biological/applied materials are not only the current hotspot but also the future development trend.

Out of the 23,556 researchers, Ma, Rong and Alifu, Nuernisha have published the most articles, followed by Yan, Ting and Alimu, Gulinigaer. Which shows that they are leading researchers in their current field.

### 4.2 Research hotspots

By analyzing the frequency of occurrence of keywords, it can reflect the hotspot of research and the trend of nanotechnology development in the application of cervical cancers. From the keyword analysis, it can be seen that the combination of nanoparticles and cervical cancer is a hot topic in current research.

#### 4.2.1 Nanoparticles in the field of cervical cancer

The increasing number of cervical cancer cases among women highlights the need to improve diagnostic methods and determine the stage of the disease. Nanotechnology has emerged as a promising field, allowing the utilization of nanomaterials in various diagnostic procedures. This utilization is driven by the unique physical characteristics of nanomaterials, which enhance the precision of diagnosis and the effectiveness of treatment. Therefore, the application of nanotechnology in the diagnosis and treatment of cervical cancer in women holds great potential to significantly improve the efficiency and accuracy of detection and therapy, providing valuable time for subsequent treatment.

##### 4.2.1.1 Role of nanotechnology in HPV virus detection

Based on empirical evidence, it has been established that high-risk variants of HPV exhibit a strong correlation with the onset of cervical cancer, as approximately 96% of cervical cancer cases are associated with these variants (22). Consequently, the precise identification of the HPV virus plays a pivotal role in both the prevention and early diagnosis of cervical cancer. Traditional methods for detecting HPV infection encompass the Pap test, visual inspection with acetic acid, and HPV serologic testing; however, these methods are hindered by their considerable expenses and limited specificity, thereby necessitating further enhancements (23). In this particular context, the utilization of nanomaterials’ exceptional properties holds promise for the development of diverse biosensors and electrosensors. These advanced sensors have the potential to enable rapid, accurate, and convenient detection of the HPV virus. Furthermore, they can enhance the sensitivity of detection by accurately identifying specific DNA and mRNA within the HPV virus. Gold nanoparticles are frequently employed in diverse sensor applications owing to their distinctive physicochemical properties, facile synthesis, surface functionalization, capabilities, and the ability to tune their optical properties based on size and shape (24); Moreover, graphene emerges as a highly efficient sensing technique for rapid detection through integration, with its functional groups demonstrating exceptional reliability in capturing molecules and analyzing their interactions with specific targets (25). Researchers have made significant progress in developing sensors using nanomaterials for HPV virus detection. One such example is the electrochemical DNA-biosensor developed by Pegah Mahmoodir et al. This biosensor utilizes the unique properties of the GO honeycomb structure, including high surface area, high electrical conductivity, and high chemical stability. Additionally, the incorporation of gold nanoparticles (AuNPs) enhances the electrical conductivity and creates immobilized channels for DNA on the electrode surface. The experiments conducted with this biosensor have demonstrated reliable results (26). In addition to genetic detection of HPV viruses, nanomaterials can also be used to detect HPV-associated proteins. Nanoparticles such as AuNPs have plasmon resonance (LSPR) properties, which allow them to be ionized under laser irradiation. This ionization generates characteristic mass reporter ions, making AuNPs suitable as mass labels for signal amplification. Additionally, by coupling antibodies to the nanoparticles, specific antigens can be targeted and marker proteins can be detected (27). Toby Siu-Chung Tam et al. utilized the property of AuNPs to develop an antibody-coupled AuNPs mass tag for the detection of HPV18E7 protein, enabling early cervical cancer diagnosis (28). Currently, the focus of nanomaterial application lies primarily on AuNPs, but there are still numerous other nanomaterials that require further study. Moreover, the combination of nanomaterials with other nanomaterials or molecules results in multifunctional nanoparticle couplings. These couplings possess both the physicochemical properties of the nanoparticles themselves and the functions of the couplings, thereby significantly enhancing the accuracy of nanoparticle diagnosis. This area of research remains a prominent and active field. With the continuous advancements in nanotechnology, there is immense potential to utilize various high-performance nanomaterials for the detection of HPV virus and the diagnosis of cervical cancer.

##### 4.2.1.2 The role of nanotechnology in the detection of tumor markers for cervical cancer

Tumor markers are molecules that indicate the presence or prognosis of malignant tumors and can be used for the early diagnosis of tumorigenesis and recurrence, as well as to determine tumor prognosis and treatment efficacy (29). Currently, the application of nanotechnology in the detection of tumor markers is mainly to combine various types of nanoparticles with sensors and use their unique physicochemical properties to improve the accuracy of detection. Common tumor markers for cervical cancer include squamous cell carcinoma antigen (SCCA) and carcinoembryonic antigen (CEA), as well as other characteristic proteins and genes. SCCA is a glycoprotein that shows significantly elevated levels in patients with cervical cancer. It is considered as one of the suitable biomarkers for the detection of cervical cancer (30). Therefore, it is crucial to identify and quantify the critical level of SCCA for effective detection and treatment of cervical cancer. In addition the serum concentration of SCCA correlates with tumor stage, treatment outcome, recurrence and survival in patients with squamous cell cervical cancer (31). Therefore, an effective detection method is of particular importance in identifying and quantifying the severity of gynecological tumors. Xinmei Liu et al. utilized the high sensitivity of gold nanoparticles coupled to analyte molecules to detect and quantify SCCA levels (32). This method has demonstrated exceptional sensitivity in experiments and significantly aided in the detection of cervical cancer. Additionally, the abnormal methylation of genes in the promoter regions of tumor suppressor genes can impede DNA transcription, which is a critical process in tumors (33). Thus, methylation of genes may also be a biomarker for cervical cancer detection. Jin Huang et al. exploited the property that the transition of AuNPs from the dispersed to the aggregated state can lead to the discoloration of colloidal solutions. They employed this characteristic to develop a novel colorimetric detection method for identifying methylation of the PAX1 gene in cervical scrapings. This was achieved by combining sulfhydryl-labeled primers with AuNPs (34). Through a clinical trial of 42 patients with cervical cancer, the study found that the percentage of normal median methylation rate detected was 33.11%, while the percentage of CIN1 type median methylation rate detected was 45.14%. Additionally, the percentage of CIN2 type median methylation rate was 74.64%, the percentage of CIN3 type median methylation rate was 67.81%, and the percentage of SCC series median methylation rate was 82.64%. These results indicate a positive correlation between the methylation status and frequency of PAX1 with the severity of cervical cancer. Moreover, the researchers suggest that this simple, fast, and inexpensive colorimetric test has the potential to enhance the efficiency of early cervical cancer screening when compared to traditional detection methods. Furthermore, nanoparticles exhibit promising potential in fluorescence imaging of cervical cancer. This is attributed to their exellent absorption and fluorescence characteristics in the near-infrared region. Consequently, further research in this area is highly recommended (35). Based on the properties of different types of nanomaterials, including electrical conductivity, optical properties, magnetic or plasma properties, the combination of nano ions with traditional detection methods offers a new approach to enhance detection sensitivity and reduce costs in the diagnosis of cervical cancer. As nanotechnology continues to advance utilization of nanomaterials for detecting cervical cancer is expected to become more widespread. These nanomaterials are anticipated to outperform traditional detection methods in terms of sensitivity and reliability, while also mitigating operational challenges and minimizing the influence of the body environment on diagnostic outcomes. We could believe that this has the potential to revolutionize cervical tumors diagnosis.

##### 4.2.1.3 Nanoparticles in photodynamic therapy

Nowadays, photodynamic therapy (PDT) has emerged as a groundbreaking treatment for cervical cancer in women. In contrast to conventional therapies like surgery, radiotherapy, and chemotherapy, which are associated with drawbacks such as extensive wounds, severe side effects, and drug resistance, PDT offers a minimally invasive approach that is resistant to multidrug resistance. This is due to its utilization of a clinical procedure that minimizes invasiveness, as well as a distinct toxicity mechanism targeting cancer cells, which sets it apart from chemotherapy (36, 37). The cytotoxic effect of PDT on cancer cells primarily relies on the accumulation of photosensitizers (PS) in target tissues. When these PS are exposed to light of specific wavelengths matching their absorption spectrum, they transition from the ground state to an unstable excited state. This process generates singlet oxygen species, which further produce other reactive oxygen species (ROS). These ROS oxidize critical components within cancer cells, leading to an acute cellular stress response and ultimately resulting in cell death (38). Designing a reliable PS is crucial for the efficacy of PDT. Nanoparticles offer several advantages, including enhanced light absorption, higher photostability, and better biocompatibility. These properties assist in stabilizing and absorbing light, while minimizing side effects on the human body. Moreover, nanoparticles possess passive targeting capabilities, allowing them to accumulate intensively in tumor tissues through the enhanced permeability and retention (EPR) effects, thereby enhancing the effectiveness of the treatment (39). In order to enhance the targeting of tumors and improve the precision of treatment while minimizing damage to normal human cells, researchers have focused on targeting the tumor environment and various markers of cervical cancer. One approach is to load specific moieties onto specific nanoparticles, which has shown promising results (40, 41). Such as taking advantage of the elevated expression of molecular proteins, such as the CD44 receptor, in the tumor microenvironment, researchers utilized hyaluronic acid (HA) coupled with chloro e44 (Ce6) to create Ce6-coupled HA nanophotosensitizers. These nanophotosensitizers were specifically designed to target HeLa human cervical cancer cells by binding to the CD44 receptor (42). In addition, in a clinical trial conducted by Antonio Carlos Figueiredo Vendette et al., 12 patients were treated with PDT, and 11 of them achieved the desired results. Among the 11 patients, two received 2 treatments while the rest received only one treatment. Importantly, none of these patients experienced any cytotoxicity, indicating that the combination of nanotechnology and PDT holds promise as a safe treatment for cervical cancer. However, it should be noted that 11 of the patients in this trial had CIN type 1, while only 1 had CIN type 2. Therefore, further investigation is needed to explore the effectiveness of PDT in treating highly differentiated cervical cancer (43). However, there are still a number of problems in the practical application of PDT in the clinic: firstly, PDT is currently mainly applied to superficial tumors in the human body, with limited effect on deep tumors where light transmission is blocked; secondly, the photosensitization of the patient’s skin after undergoing PDT is susceptible to side-effects; and lastly, due to the hydrophobicity of PS and the oxygen-dependence of PDT, the potential of PDT for the treatment of cervical cancer is currently limited (44–47). After all, with the continuous advancement of nanotechnology, incorporating nanoparticles into PS may help overcome these challenges faced by PDT in clinical settings.

#### 4.2.2 Nanotechnology in the drug delivery of cervical cancer

The mechanism of nanotechnology in drug delivery for cervical cancer treatment involves the preparation of nanoscale drug carriers. These carriers encapsulate anticancer drugs and deliver them accurately to cervical cancer tumor cells through targeted delivery and release. By altering the surface properties and structure of nanodrug carriers, they can achieve targeted delivery and better interact with cancer cells. Once the nanodrug carrier reaches cancer cells, it gradually releases the drug into the tumor cells through its own special structure or drug release mechanism. This targeted and controlled release method improves drug efficacy, reduces side effects, and minimizes drug resistance during treatment. The application of nanotechnology offers more precise and effective drug delivery methods for the treatment of cervical cancer.

By altering the size, shape, and surface properties of drug carriers, nanotechnology has the potential to enhance the precision of drug delivery (48, 49). Chemotherapy is currently the primary treatment for tumors, but its efficiency is hampered by low solubility and lack of selectivity, which can increase toxicity to normal cells. Therefore, there is a need to focus on studying excellent carriers or developing new treatment strategies to address these issues and improve the effectiveness of chemotherapy (39, 50). To overcome the limitations of chemotherapeutic agents, various synergistic therapeutic systems have been developed based on the characteristics of the tumor microenvironment. One such system is a targeted redox-sensitive micellar system (DOX/FCH) composed of ferrocene (Fc) and hyaluronic acid (HA), which enables the delivery of doxorubicin (DOX) for synergistic chemotherapy and chemodynamic therapy (CDT). The high affinity between HA and the CD44 receptor, which is highly expressed in human cervical cancer (HeLa) cells, allows DOX/FCH to accurately target the tumor site. The appropriate particle size facilitates the absorption of micelles by cells. Under a reducing environment that mimics the intracellular conditions of tumor cells, disulfide bonds are depolymerized, leading to approximately 50% release of DOX from DOX/FCH within 2 hours. The good biocompatibility of FCH in 3T3 cells and its evident cytotoxicity in HeLa cells suggest that FCH holds great potential as a nanocarrier. In addition, the cytotoxicity of DOX/FCH confirms the synergistic effect of CDT and DOX chemotherapy with FCH. Hence, DOX/FCH shows promising prospects for enhancing the efficacy of combination chemotherapy and CDT (51).

Nanotechnology has the potential to safeguard medications from the effects of the external environment and enhance their durability. For instance, in a specific research study, nanoparticles were used to encase the chemotherapy medication cisplatin, effectively preventing premature decomposition and metabolism within the body. This prolonged the drug’s duration of action and improved its therapeutic efficacy (52). Small molecule induced DNA hydrogels with encapsulation and release properties are also effective in maintaining drug properties. DNA, a natural biopolymer, can assemble polyA tail DNA motif into hydrogel using small molecule cyanuric acid. Researchers have conducted experiments on the encapsulation of various substances, including a small chemotherapy drug, a fluorescent molecule, two proteins, and several nanoparticle formulations. The results demonstrated the release of doxorubicin, small fluorescent molecules, and fluorescent labeled antibodies (53).

Nanotechnology can also enhance the permeability of drugs, allowing them to traverse blood-tissue or tumor tissue barriers and access cervical cancer lesions. In a particular study, doxorubicin, a chemotherapy medication, exhibited improved permeability in cervical cancer lesions, leading to a significant enhancement in its anti-tumor efficacy (54). Due to the enhanced permeation and retention effects, stereocomplex micelles can selectively accumulate at the tumor site. In this study, a reliable drug delivery mechanism was created by combining enantiomeric 4-armed poly (ethylene glycol)–poly (D-lactide) and poly (ethylene glycol)–poly (L-lactide), aiming to regulate drug release and enhance tumor cell absorption for effective treatment of cervical carcinoma. The DOX-loaded micelles, including poly (D-lactide)-based micelle (PDM/DOX), poly (L-lactide)-based micelle (PLM/DOX), and stereocomplex micelle (SCM/DOX), all had sizes around 100 nm, which is suitable for the augmented permeability and retention (EPR) phenomenon. The effectiveness of DOX-loaded micelles, particularly SCM/DOX, in inhibiting tumors was confirmed in the U14 cervical carcinoma mouse model. The groups treated with DOX-loaded micelles, especially the SCM/DOX group, showed a significant increase in tumor cell death and necrosis. Moreover, these DOX-infused micelles demonstrated a remarkable reduction in DOX’s systemic toxic effects. Therefore, SCM shows potential as an effective drug delivery mechanism for future treatments of cervical carcinoma (55).

Nanotechnology can be used to transport medications more efficiently to cervical cancer lesions, reducing the accumulation of drugs in healthy tissues and minimizing their adverse effects. For example, in a specific study, researchers utilized nanotechnology to encapsulate the chemotherapy medication doxorubicin within lipid nanoparticles, which helped mitigate the drug’s harmful effects on healthy tissues such as the liver (56). Moreover, novel epirubicin-loaded nanoformulations were prepared from doxorubicin, which has a higher biocompatible therapeutic index (57). Cancer-specific drug delivery not only prevents adverse effects but also enhances drug accumulation in tumors. Therefore, surface modification of targeted selenium nanoparticles (SeNPs) with folic acid (FA) is an effective strategy for cancer treatment. FA-SeNPs nanoparticles are created by modifying SeNPs with folic acid, which binds to receptors overexpressed on the surface of cancer cells, including human cervical cancer HeLa cells, resulting in a tumor-targeting delivery vehicle. Subsequently, the anticancer drug DOX is loaded onto the surface of FA-SeNPs to improve its anti-tumor efficacy in the treatment of human cervical cancer (58).

Moreover, nanotechnology has the potential to create a combination of multiple drugs. Fytas et al. (Year) synthesized novel 1-(2-aryl-2-adamantyl)piperazine derivatives and evaluated their antitumor properties against HeLa cervical cancer *in vitro* (59). Chen et al. (Year) investigated the improvement of cancer treatment through the use of an acid-reactive cytotoxic peptide-doxorubicin compound (60). Nanotechnology has the potential to unite multiple drug carriers, enabling the implementation of multidrug combination therapy, which enhances therapeutic effectiveness and reduces tumor resistance to individual medications.

In the past time, although researchers have done many experiments on nanoparticles in drug delivery, the efficiency of translating research results into clinical applications is still low (61). Most of the applications of nanomedicines in cervical cancer are still in the laboratory stage. Therefore, it is becoming increasingly important to translate effective nanoparticle delivery methods from the experimental stage to clinical applications.

In conclusion, nanotechnology has the potential to enhance drug targeting, improve drug stability, increase drug permeability, and reduce drug side effects in the treatment of cervical cancer. This technology could offer a novel approach to treating cervical cancer.

### 4.3 Future trend

The prominence graph of a keyword shows how long a certain keyword stays popular. The prominence graph allows us to obtain the possible trend in the future. From the **Figure 4B**, we can learn that photothermal therapy, Indocyanine Green, etc may be a new direction for future research.

#### 4.3.1 Photothermal therapy

**Figure 5:**
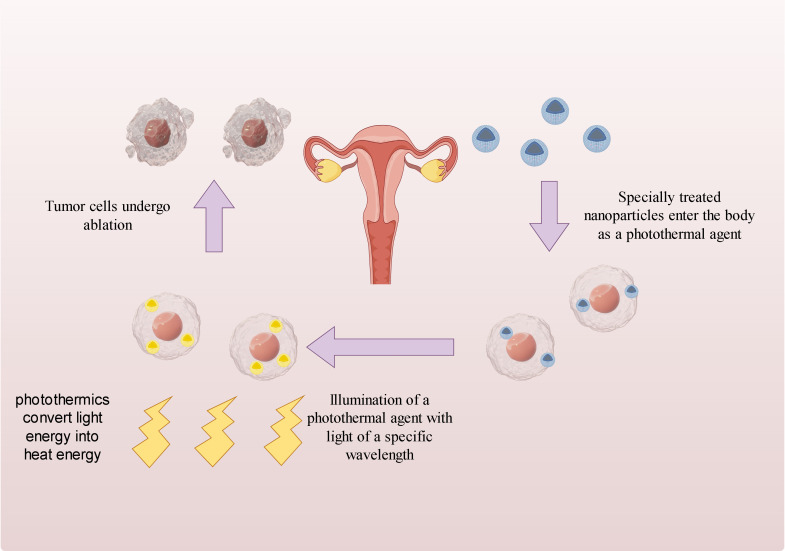
Mechanism of action of photothermal therapy.

Despite radiotherapy being the primary treatment for cervical cancer, its potential side effects can significantly impact the outlook for patients. Recently, there has been a growing focus on near-infrared laser-induced photothermal therapy due to its advantageous biological safety characteristics (62). This therapy involves using light of a specific wavelengthto heat a photothermal agent, effectively killing tumor cells. **Figure 5** demonstrates how nanoparticles are internalized in cells and convert light energy into heat, leading to the death of cancer cells. Photothermal therapy not only eliminates local tumor cells and minimizes damage to normal tissue cells, but also avoids the development of drug resistance, making it more effective than traditional radiotherapy. Nanotechnology advancements have enabled various nanomaterials to enhance the effectiveness of photothermal therapy and reduce its adverse effects through superior light absorption, conversion capacity, and biocompatibility. Moreover, certain nanomaterials can serve as both photothermal converters and innovative radiosensitizers, integrating radiotherapy and improving patient prognosis by enhancing photoelectric absorption efficiency and electron presence (63, 64). Apart from serving as photothermal converting agents for photothermal therapy, nanoparticles of appropriate size can effectively guide drugs or photothermal agents to the desired tumor site due to their increased permeability and retention effect (65). This nanomaterial-based transport approach has the potential to significantly enhance treatment precision and effectiveness, while minimizing harm to healthy tissue cells. In addition to passive targeting, magnetic targeting may emerge as a promising technique in clinical applications (66). Magnetic targeting technology relies on external conditions to enhance the accumulation of nanoparticles in the body, without altering the internal conditions, and has the capability to effectively eliminate tumor tissues (67). Currently, most experiments in this field are limited to *in vitro* cellular experiments or *in vivo* experiments in mice, with limited clinical data. However, nanoparticles have demonstrated promising results in both *in vitro* and ex vivo experiments. The application of nanotechnology in clinical photothermal therapy requires further investigation. Despite the recognition of nanoparticles’ potential in photothermal therapy, there is still much room for exploration. In the future, the focus may shift towards discovering nanomaterials that are safer, more precise, and more efficient, while also increasing the sensitivity of tumor cells to radiotherapy.

#### 4.3.2 Indocyanine green

Indocyanine Green (ICG) is a fluorescent dye commonly used in clinical practice and has been studied for the treatment of cervical cancer in recent years. Its application mechanism can be divided into two aspects:

##### 4.3.2.1 Photothermal therapy

ICG exhibits good photosensitive properties and can absorb specific wavelength laser light sources. When exposed to a laser light source, ICG undergoes a photothermal effect, resulting in an increase in local temperature and causing thermal damage to cervical cancer cells. This therapy can induce tumor cell apoptosis, necrosis, and inhibit tumor growth.

For instance, researchers have explored the use of ICG-loaded lipid nanoparticles to induce intracellular thermal damage for effective treatment of cervical cancer (68). Additionally, polydopamine encapsulated novel indocyanine green therapeutic diagnostic nanoparticles have shown promise in enhancing the photothermal therapy of cervical cancer Hela cells (69–71). The use of nanomaterials with high photothermal conversion efficiency and excellent biocompatibility can significantly improve the overall photothermal conversion efficiency and increase the accumulation of photoacoustic agents in tumors (72).

##### 4.3.2.2 Fluorescence guided surgery

**Figure 6:**
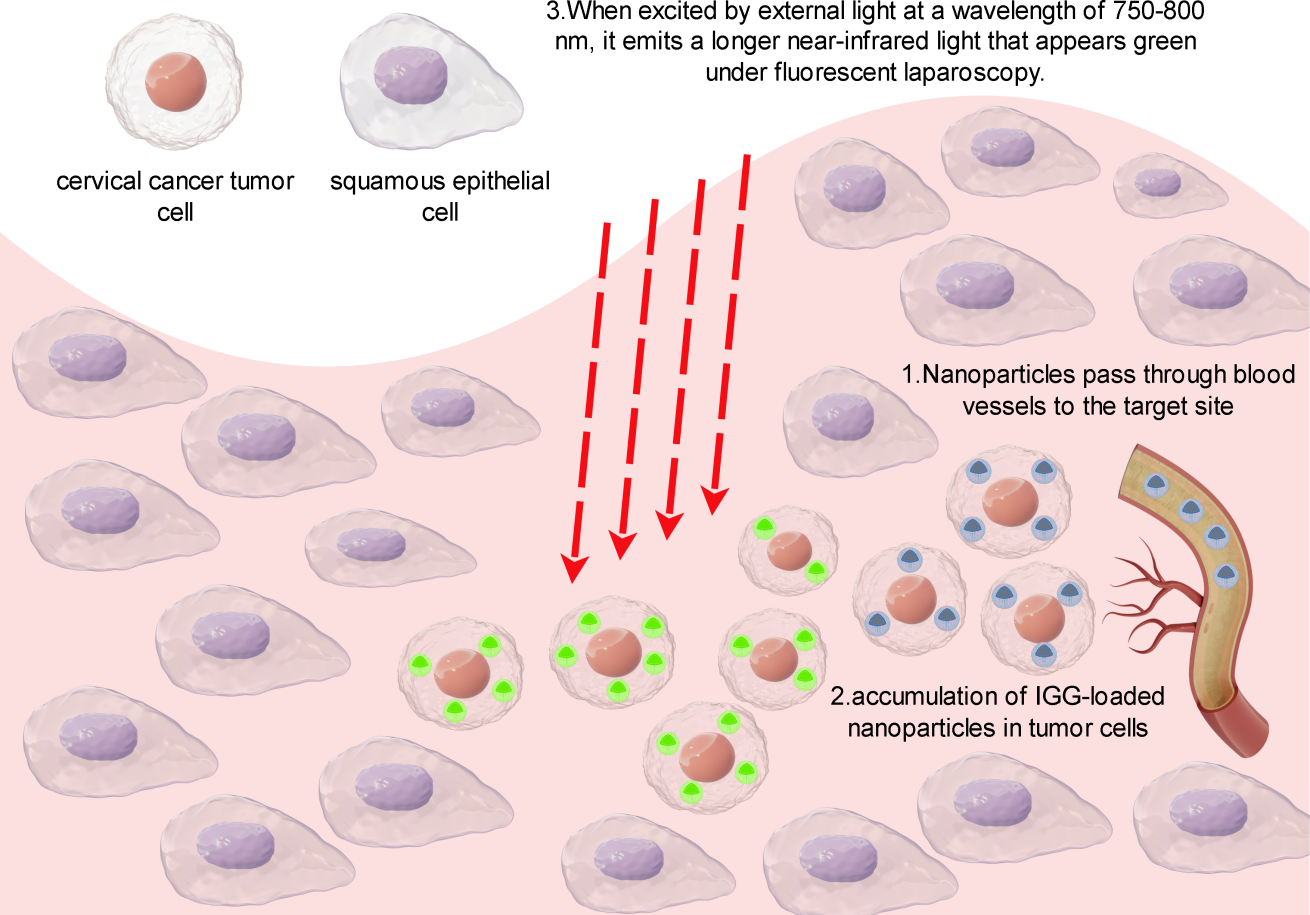
Principles of IGG-guided fluorescence imaging.

ICG exhibits strong fluorescence characteristics and can emit fluorescence under near-infrared spectroscopy. It is a near-infrared fluorescent dye that is biocompatible. When excited by external light with a wavelength of 750-800 nm, it emits longer wavelength near-infrared light, appearing green under fluorescence laparoscopy. In cervical cancer surgery, doctors can intravenously inject ICG into patients, taking advantage of its accumulation in tumor tissue to emit strong fluorescence signals under near-infrared spectroscopy. Fluorescence guided surgery can then be used by doctors to locate and remove tumors, enhancing the accuracy and thoroughness of the procedure. According to **Figure 6**, it can be observed that ICG-loaded nanoparticles accumulate in tumor cells after entering the body through blood vessels. Subsequently, when these tumor cells are exposed to infrared light, they exhibit a green fluorescence in the laparoscope.

For instance, ICG dye can be utilized for sentinel lymph node (SLN) mapping in cervical cancer. SLN mapping is a viable surgical strategy that determines the necessity for radical lymph node dissection. It is based on the concept that SLN serves as the initial site of lymph node metastasis. If SLN is unaffected, it is theoretically assumed that the lymph nodes behind SLN should also be negative. The postoperative pathological confirmation rate of lymph node metastasis in most cervical cancer patients is approximately 24.16% (73). Therefore, pelvic lymph node dissection would not benefit them. The purpose of SLN biopsy is to avoid over-treatment by performing complete pelvic lymph node dissection on negative lymph nodes. This approach helps prevent complications associated with radical lymphadenectomy (74).

Combining indocyanine green (ICG) with nanoparticles can improve imaging localization. The use of ICG loaded with hyaluronic acid conjugated lactide-co-glycolide nanoparticles (HINPs) can enhance the target specificity of cervical cancer tumors. *In vivo* studies have demonstrated that HINPs enable near-infrared fluorescence imaging in cervical cancer cell lines. The efficiency of HINPs in delivering *in vivo* and the efficiency of free ICG in cervical cancer tumors were compared. Overall, HINPs have the potential to enhance NIR fluorescence image-guided surgery by assisting in the visualization of CD44-positive cervical cancer (75).

ICG imaging of lymph nodes is non-specific and cannot accurately distinguish between metastatic lymph nodes and inflammatory lymph nodes, resulting in a high false positive rate for tumor metastasis imaging. Additionally, ICG has inherent limitations such as poor water stability and low penetration depth, which affect its imaging performance. Furthermore, the lack of reactive groups in ICG makes it challenging to chemically synthesize it with other agents, such as targeting peptides. To overcome these limitations, active targeting using tumor-targeting ligands, such as targeting peptides, can be achieved by modifying the surface of nanoparticles. In this regard, the combination of the tumor metastasis-targeting peptide TMTP1 and ICG-loaded poly (ethylene glycol)-poly(lactide-co-glycolide acid) (PEG-PLGA) micelles was used to achieve active targeting. PEG-PLGA is a promising biodegradable polymer known for its biocompatibility, biodegradability, and sustained drug release properties, which contribute to more accurate imaging (76).

In summary, ICG can play a role in cervical cancer through two mechanisms: photothermal therapy and fluorescence guided surgery. Photothermal therapy utilizes the photosensitive properties of ICG to generate thermal effects and kill tumor cells through light irradiation. Fluorescence guided surgery utilizes the fluorescence characteristics of ICG to help doctors accurately locate and remove tumors during surgery, improving surgical effectiveness.

### 4.4 Limitation

The literature in our study may not be comprehensive. Firstly, our study focused only on WOOSC data and excluded data from other major search engines such as PubMed, EMBASE, and OVERE. Additionally, there is a language bias as we only retrieved articles published in English. Therefore, the articles found may not fully represent the body of research related to nanotechnology and cervical cancer. Secondly, high-quality papers published recently might not have received adequate attention due to low citation rates. These findings highlight the importance of regularly updating research. Although publications beyond September 20th, 2023 were excluded due to insufficient information, this review includes the majority of articles published between 2014 and 2023 in the field of cervical cancer and nanotechnology. Therefore, new data is unlikely to significantly impact the final results.

## 5 Conclusions

After conducting a comprehensive analysis using various scientometric instruments, we have successfully characterized the nanotechnology literature in the field of cervical cancer. Additionally, we have reviewed the current trends in nanotechnology development in cervical cancer-related fields and identified the current research hotspots. Moreover, we have analyzed the potential future directions for research in this area. The continuous growth of literature in this field signifies its increasing global importance, with China emerging as the leading publisher in this domain. The current research hotspots revolve around ‘nanoparticles’, ‘cervical cancer’, and ‘drug delivery’, while the future research trend may involve the integration of nanotechnology with ‘photothermal therapy’ and ‘indocyanine green’. It is evident that further research is required to fully explore the potential applications of nanotechnology in cervical cancer. The findings of these studies can provide valuable insights for new researchers in this field and contribute to the advancement of cervical cancer therapy.

## Data availability statement

The raw data supporting the conclusions of this article will be made available by the authors, without undue reservation.

## Author contributions

XS: Writing – original draft. XL: Writing – original draft. ZT: Writing – review & editing. LZ: Writing – review & editing.

## Supplementary material

Supplementary Figure 1
**(A)** Author co-authorship analysis carried on CiteSpace. **(B)** Analysis of co-cited authors carried on CiteSpace.

Supplementary Figure 2 The collaboration network of institutions visualization map.
