# Corrigendum: Recent status and trends of nanotechnology in cervical cancer: a systematic review and bibliometric analysis

**DOI:** 10.3389/fonc.2024.1395166

**Published:** 2024-03-21

**Authors:** Xiangzhi Song, Xun Li, Zhiwei Tan, Lushun Zhang

**Affiliations:** ^1^ School of Clinical Medicine, Chengdu Medical College, Chengdu, China; ^2^ Department of Pathology, the Second Affiliated Hospital of Chengdu Medical College, China National Nuclear Corporation 416 Hospital, Chengdu, Sichuan, China; ^3^ Development and Regeneration Key Laboratory of Sichuan Province, Department of Neurobiology, Chengdu Medical College, Chengdu, China; ^4^ Department of Pathology and Pathophysiology, Chengdu Medical College, Chengdu, China

**Keywords:** nanotechnology, cervical cancer, bibliometrics, nanoparticles, drug deliver

In the published article, there was an error. We have improperly phrased the first sentence of the abstract’s background and the first sentence of the article’s introduction.

Corrections have been made to Abstract and Introduction. These sentences previously stated:

(1) “Cervical cancer is currently the second leading cause of death among women.”
(2) “Globally, cervical cancer is the second most common type of cancer among women and the fourth leading cause of cancer-related deaths among women.”

The corrected sentences appear below: “Cervical cancer is currently the second leading cause of cancer death among women from developing countries (1).”; “Globally, cervical cancer is the fourth most common type of cancer among women and the second leading cause of cancer-related deaths among women from developing countries (1, 2).”

The authors apologize for these errors and state that this does not change the scientific conclusions of the article in any way. The original article has been updated.
