# Supplementary material for: Recent status and trends of nanotechnology in cervical cancer: a systematic review and bibliometric analysis
Source: Front Oncol. 2024 Feb 20;14:1327851. doi: 10.3389/fonc.2024.1327851 (PMC10912161; doi:10.3389/fonc.2024.1327851)
Supplement: Supplementary file 1 [file DataSheet_1.docx]

((TS=(nanotechnology or Nanomedicine or nanoparticles or drug deliver)) AND TS=(Uterine Cervical Neoplasms or Cervical Neoplasms or cervical cancers or Cervix Cancers or Uterine Cervical Cancer or Cancer of the Cervix))
