# Supplementary figures and images for: Recent status and trends of nanotechnology in cervical cancer: a systematic review and bibliometric analysis
Source: Front Oncol. 2024 Feb 20;14:1327851. doi: 10.3389/fonc.2024.1327851 (PMC10912161; doi:10.3389/fonc.2024.1327851)

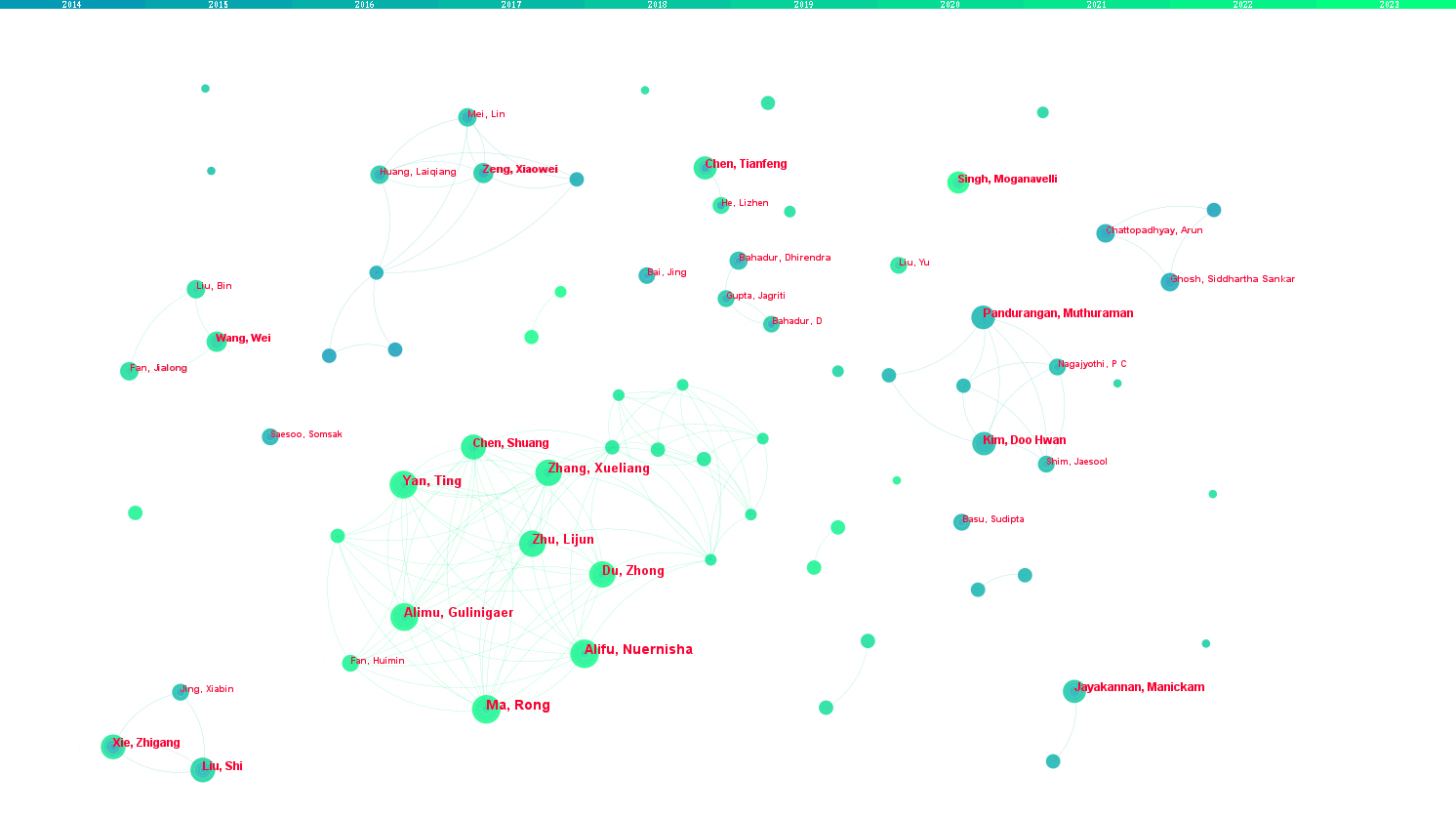

Supplement: Supplementary Figure 1 — (A) Author co-authorship analysis carried on CiteSpace. (B) Analysis of co-cited authors carried on CiteSpace. [file Image_1.jpeg]

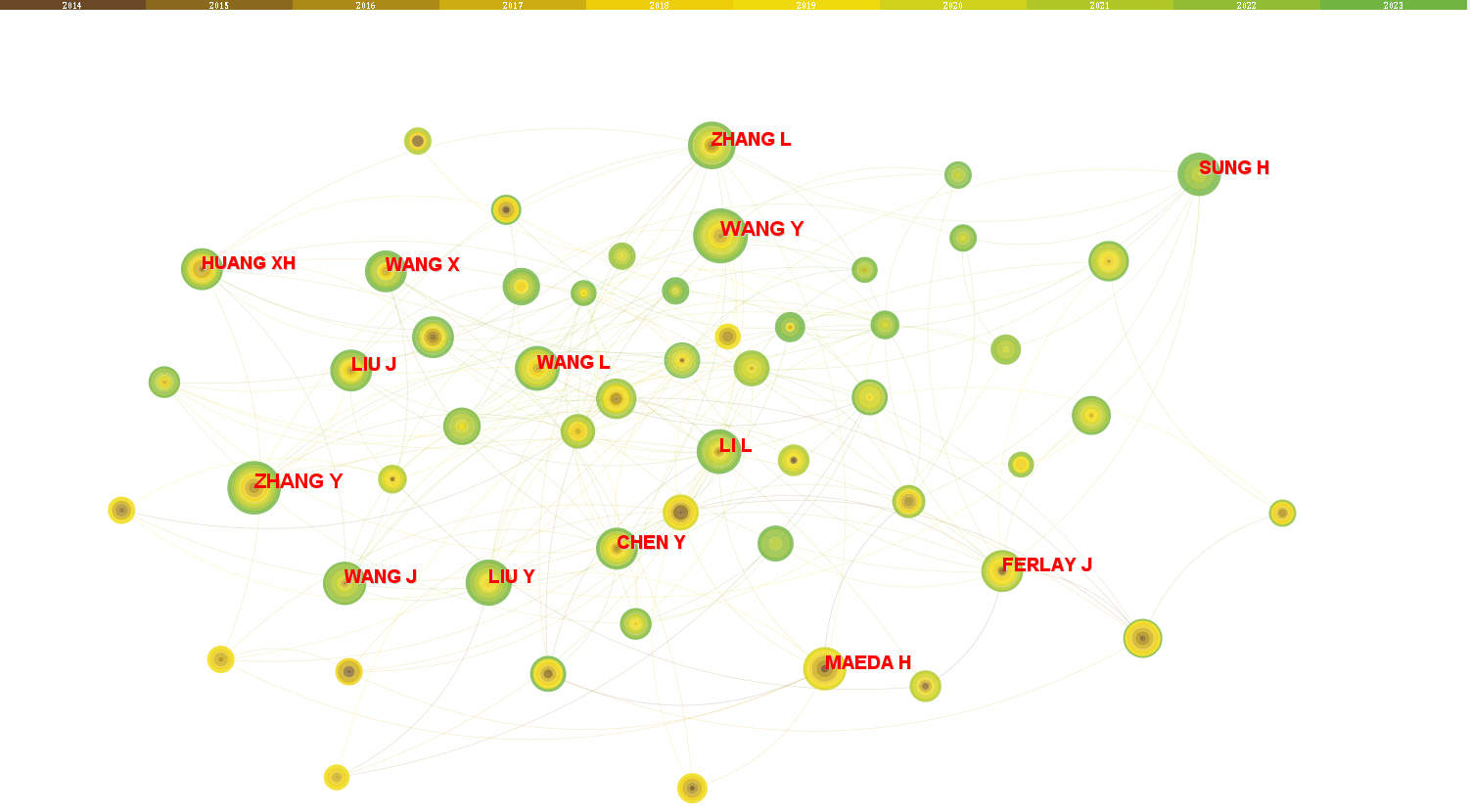

Supplement: Supplementary Figure 2 — The collaboration network of institutions visualization map. [file Image_2.jpeg]

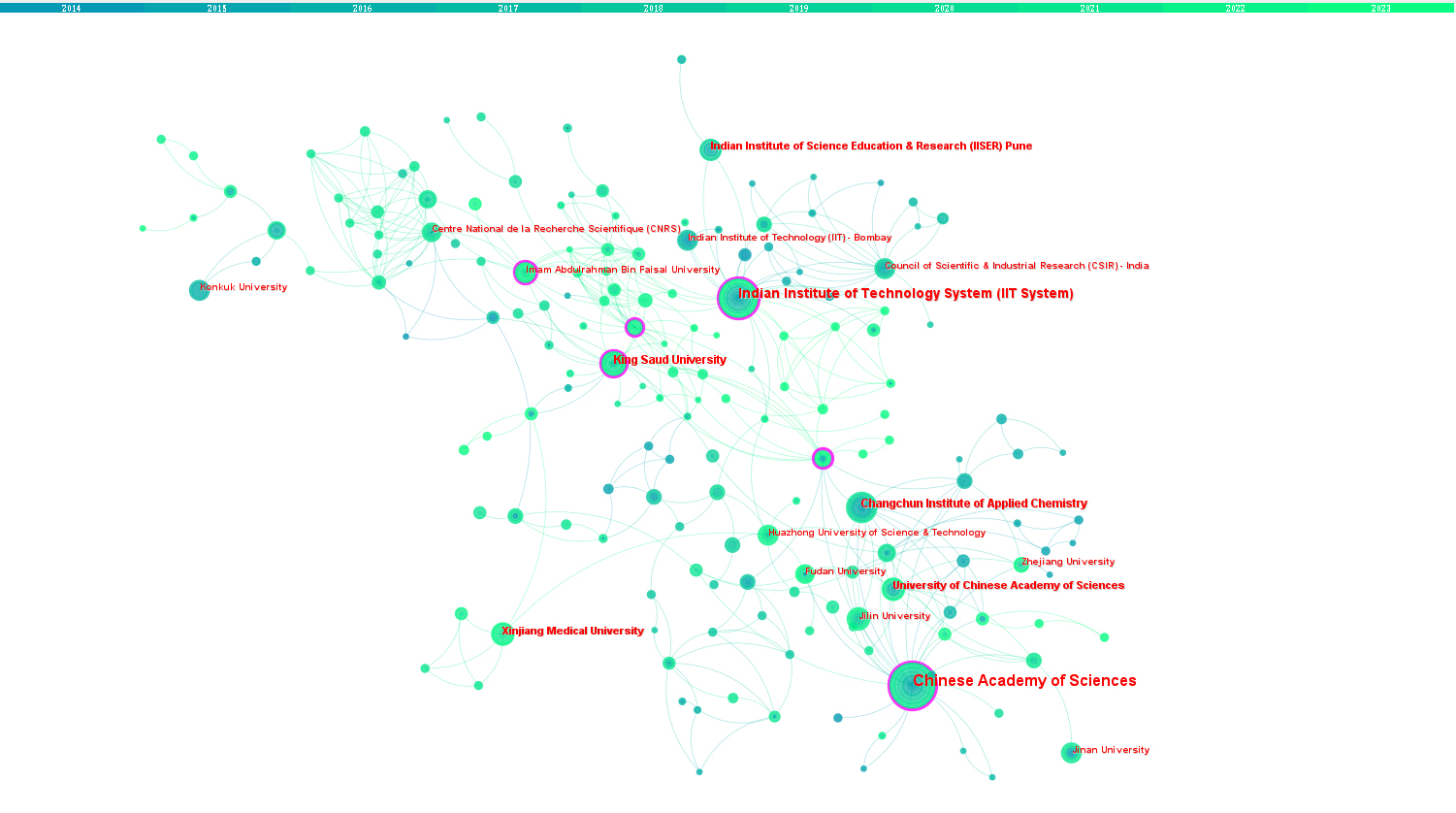

Supplement: Supplementary file 4 [file Image_3.jpeg]
