# Supplementary material for: Recent status and trends of nanotechnology in cervical cancer: a systematic review and bibliometric analysis
Source: Front Oncol. 2024 Feb 20;14:1327851. doi: 10.3389/fonc.2024.1327851 (PMC10912161; doi:10.3389/fonc.2024.1327851)
Supplement: Supplementary file 5 [file Table_1.docx]

**Supplementary material**

**Supplementary Table 1. Top 20 keywords with related fields.**

| Rank | Count | key word |
| --- | --- | --- |
| 1 | **295** | **nanoparticles** |
| 2 | **248** | **cervical cancer** |
| 3 | **218** | **drug delivery** |
| 4 | **143** | **in vitro** |
| 5 | **131** | **delivery** |
| 6 | **104** | **apoptosis** |
| 7 | **102** | **cells** |
| 8 | **98** | **gold nanoparticles** |
| 9 | **97** | **therapy** |
| 10 | **92** | **cancer** |
| 11 | **77** | **release** |
| 12 | **72** | **doxorubicin** |
| 13 | **63** | **cytotoxicity** |
| 14 | **59** | **silver nanoparticles** |
| 15 | **56** | **green synthesis** |
| 16 | **55** | **human papillomavirus** |
| 17 | **52** | **toxicity** |
| 18 | **52** | **photodynamic therapy** |
| 19 | **51** | **size** |
| 20 | **50** | **chemotherapy** |
